# Snake-Like Robot Workspace Solving Method Based on Improved Monte Carlo Method

**DOI:** 10.1155/abb/6125695

**Published:** 2025-03-09

**Authors:** ZhiYong Yang, Wang Tian, HaoYang Wang, Xu Liu, DaoDe Zhang, Yu Yan, ShaoSheng Fan

**Affiliations:** ^1^Hubei Key Laboratory of Modern Manufacturing Quantity Engineering, School of Mechanical Engineering, Hubei University of Technology, Wuhan, Hubei 430068, China; ^2^State Grid Hunan Ultra High Voltage Transformer Company, Transformer Intelligent Operation and Inspection Laboratory, Changsha, Hunan, China; ^3^School of Electrical Engineering, Changsha University of Science and Technology, Changsha, Hunan, China

**Keywords:** α-shape, Monte Carlo method, snake-like robot, volume calculation, workspace

## Abstract

The method is applicable for solving the obstacle avoidance workspace of a snake-like robot working on high-voltage transmission cables, based on an improved Monte Carlo method, to address the issues of uneven distribution of scattered points, difficulty in extracting point cloud boundaries, and insufficient accuracy in traditional Monte Carlo methods. The proposed method first generates a seed workspace for the snake-like robot using traditional Monte Carlo method and then envelops the seed workspace with a cube and divides it into several smaller cubes that contain points in the workspace equally. Next, Gaussian distribution probability density function is used to extend and sample the seed workspace of the robot, generating the workspace of the snake-like robot. Finally, the *α* − shape algorithm is used to extract the point cloud boundaries of the snake-like robot workspace and calculate its volume, accurately determining the workspace. Simulation experiments comparing the reconstructed surface obtained from the *α* − shape algorithm with the point cloud of the snake-like robot workspace show high accuracy.

## 1. Introduction

Snake-like robots, as a type of specialized robot that can be used in various special operational environments such as narrow pipelines, underground caves, internal structures of buildings, underwater oil and gas pipelines, nuclear reactors, and other complex and dangerous environments, possess high redundancy and multiple degrees of freedom. These characteristics enable them to perform tasks that general-purpose industrial robots cannot accomplish and to conform to curved paths in space [1–4]. Overhead high-voltage transmission cables are often located in harsh outdoor environments such as high mountains, large rivers, and forests, where transportation is inconvenient and manual inspections are strenuous and inefficient. Most traditional inspection robots have rigid structures with two or three arms, which are suitable for working in specific tower environments. Utilizing the redundant degrees of freedom of snake-like robots allows them to adapt to various types of line environments. Applying snake-like robots to the inspection tasks along high-voltage transmission lines, replacing traditional manual inspections, is a beneficial extension of the application of snake-like robots.

As a type of redundant robot, snake-like robots typically consist of three main components: mechanism, control system, and drive system [5, 6]. After decades of development, numerous research achievements have been produced by domestic and international research institutions, with the majority of them focusing on the control system [7–9], while research on the mechanism [10] and drive system [11] is relatively limited. However, the robot's configuration directly determines whether the robot has a closed-form inverse solution and whether it has enough flexibility to adapt to different working conditions and environments [12]. Therefore, the configuration research of snake-like robots is crucial to determining whether they can meet practical application needs. Due to limitations in structural parameters and joint angle ranges, the actual operational workspace of the robot is nonexistent [13]. The flexible workspace [14, 15] is an approximation of the dexterous workspace and can be used for optimizing the robot's configuration.

So far, there are roughly three methods for solving the workspace of a robotic system: analytical method, geometric method, and numerical method [16]. The analytical method uses differential geometry to calculate the singular surfaces based on the mapping relationship between the workspace boundary and the singularity of the velocity Jacobian matrix [17]. Perturbation method and optimization theory are used to determine whether the singular surfaces are the boundaries of the workspace and finally calculate the boundary surfaces. The disadvantage of this method is that the solving process is complex and not generally applicable. Although the geometric method is simple and intuitive, it becomes very complicated and difficult to understand as the degrees of freedom of the robot increase, making it challenging to express the three-dimensional motion state of the robot in space [18].

Compared to the above two methods, numerical method is more universal, among which the Monte Carlo algorithm [19, 20] based on random probability sampling is a convenient and practical numerical analysis method that is almost universally applicable. It is often used to solve mathematical problems with stochastic characteristics [21] and therefore commonly employed in the workspace analysis of redundant robotic arms.

However, the accuracy of the robot workspace obtained by the Monte Carlo method depends on the number of randomly sampled points [22]. When the number of sampled points is insufficient, it cannot generate an accurate workspace, and the boundaries of the generated workspace have significant “noise” and are not smooth. By continuously increasing the number of randomly sampled points, although it can improve the situation where there are insufficient points used at the workspace boundary, most of the sampling points still appear in non-workspace boundary areas [23, 24], resulting in a waste of sampling points. In addition, the coordinate transformation equations in the forward kinematics of the robot [25, 26] are nonlinear, and the joint angle values obtained by Monte Carlo method follow a uniform distribution [27]. After mapping from joint space to operational space, the distribution of workspace points obtained will no longer satisfy a uniform distribution in the entire workspace [28]. This can result in issues with nonuniform point cloud density [29, 30] in certain areas. Areas with low point cloud density often occur at the boundaries of the workspace, leading to inaccurate workspace boundaries [31]. At the same time, areas with high point cloud density may exhibit “redundancy,” resulting in significant waste of computational resources.

In summary, this article introduces a snake-like robot with high flexibility and redundancy for operations along high-voltage transmission cables. The workspaces of the snake-like robot are solved using three different methods: traditional Monte Carlo method, *β* probability density distribution function-based method, and Gaussian probability density distribution function-based method. The results obtained from these three methods are compared, and the optimal workspace solution for the snake-like robot is determined. The precise workspace can be used for performance evaluation and trajectory planning of the snake-like robot, laying the theoretical foundation for achieving autonomous operations along high-voltage transmission cables.

## 2. Snake-Like Robot Structure and Kinematic Model

### 2.1. Snake-Like Robot Structure

**Figure 1:**
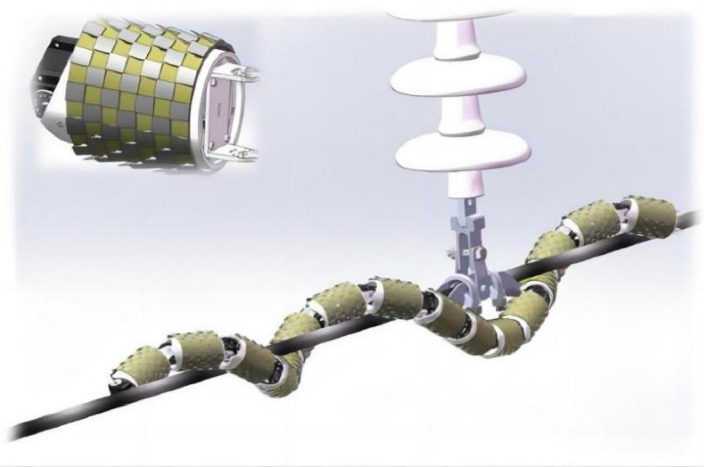
Snake-like robot obstacle traversal diagram.

This article takes the example of a snake-like robot that crawls and climbs along high-voltage transmission cables. The snake-like robot is composed of mechanical joints with high output torque, large reduction ratio, and short joint length. It features a compact structure, high load capacity, and good interchangeability. Please refer to Figure 1 for illustration. The robot is composed of several identical “P–R–S” unit modules connected in series, with each unit module having three degrees of freedom: bending (P), rotation (R), and translation (S). The bending and rotation movements allow the snake-like robot to adjust its posture in three-dimensional space. During straight segments, the robot maintains a coiled configuration with a constant pitch. When encountering obstacles, the robot wraps around the obstacles in a specific configuration while maintaining the coiled configuration in other straight segments. Mobile mechanisms are used to mimic the relative sliding mechanism of a snake's belly scales against its skeleton. In this mechanism, scale sleeves move back and forth, utilizing the antireverse characteristics of the scales to provide propulsion for forward motion.

**Figure 2:**
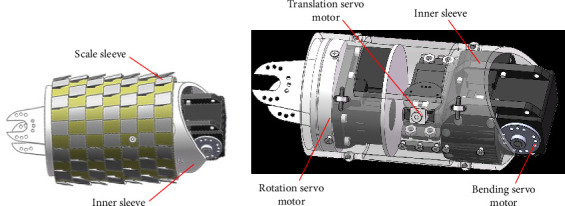
Snake-like robot unit module: (a) unit external structure and (b) unit internal structure.

The “P–R–S” unit module of the snake-like robot consists of an outer sleeve and an inner sleeve, as well as the deviation servo component, reciprocating translation servo component, and rotation servo component installed inside the inner sleeve, as shown in Figure 2. There are eight roller components fixed on the inner sleeve, which come into contact with the inner side of the scale sleeve and are mounted on the outer surface of the inner sleeve. The inner sleeve wall is equipped with slots, and the connecting component between the inner and outer sleeves of the reciprocating translation servo component, which is connected to the scale sleeve, can move back and forth within the slots to provide forward motion for the snake-like robot. During the process of adjusting the posture of the snake-like robot, the leading unit does not require deviation, and the trailing unit does not require rotation, so the leading unit does not have a deviation servo component, and the trailing unit does not have a rotation servo component.

### 2.2. Establishment of Coordinate System for Snake-Like Robot

**Figure 3:**
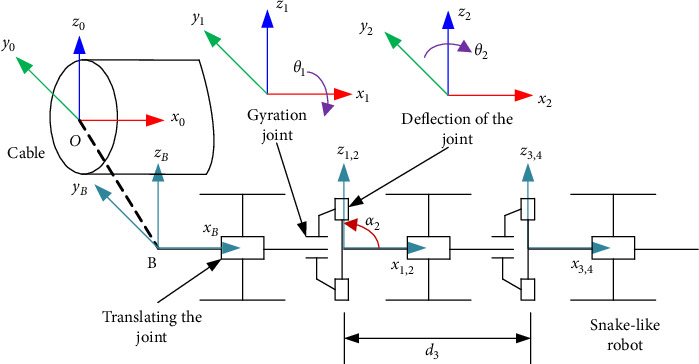
Simplified diagram of coordinate systems for snake-like robot.

The snake-like robot studied in this paper is a redundant serial robot with a special structure. In order to describe the relationship between different coordinate systems more conveniently, the following method for establishing coordinate systems will be adopted to describe the motion of the snake-like robot: Based on the characteristics of the snake-like robot's operating environment, a base coordinate system {*B*} and individual joint coordinate systems {*i*} will be established. The simplified diagram of the coordinate systems is shown in Figure 3. In the diagram, the coordinate system {*O*} represents the global coordinate system. The origin of this coordinate system is fixed on the track and does not move. It is established to obtain the mapping relationship between each joint coordinate system and the global coordinate system for kinematic analysis. The base coordinate system {*B*} is rigidly attached to the robot. The origin of the {*B*} coordinate system is located at the end of the snake-like robot unit axis, and the *X*-axis coincides with the axis of unit 1. The coordinate system {2*k*−1} represents all rotation joint coordinate systems. The origin of this coordinate system coincides with the center of rotation of the joint 2*k*−1 in the same unit, and the rotation joint rotates around the *X*-axis. The coordinate system {2*k*} represents all bending joint coordinate systems. This coordinate system is established at the center of joint 2*k*, and the bending joint rotates around the *Z*-axis.

According to Figure 3, the kinematic parameters are obtained as follows: the link length *a*_*i*_ is the distance along the *Z*_*i*_-axis from *X*_*i–*1_ to *X*_*i*_, which is the distance along the common normal. The link twist angle *a*_*i*_ is the angle between *Z*_*i*_ and *X*_*i*_ in the plane perpendicular to *a*_*i*_. The link offset *d*_*i*_ is the relative position between two links, *l*_unit_, which is the length of one unit module of the snake-like robot. The joint angle *θ*_*i*_ is the angle between two links, which can take values within the range of motion of the snake-like robot's bending joint. Based on the established kinematic model of the snake-like robot, Table 1 gives the kinematic parameters of the robot.

As the content studied in this article does not involve the reciprocating translation of the snake-like robot's steering gear, the translational variables of the robot are not considered when analyzing the kinematic problems of the snake-like robot. Only the rotation and deviation motion parameters are considered.

### 2.3. The Kinematic Model of the Snake-Like Robot

The transformation matrix between each joint coordinate system {*K*} and the global coordinate system {*O*}, as well as the transformation matrix of joint coordinate system {*K*} relative to the global coordinate system {*O*}, can be obtained from the coordinate system established by Figure 3. Let the length of each unit of the snake-like robot be denoted as *l*_unit_, and *m*^*n*^*T* represent the transformation matrix of coordinate system {*m*} relative to coordinate system {*n*}.

When *K* = 1, the coordinate system of joint 1 is obtained relative to the base coordinate system {*B*} through the following transformation: first, the coordinate system {1} overlaps with the coordinate system {*B*} initially, then moves a distance of *l*_unit_ relative to the *X*-axis of the coordinate system {1}, and finally rotates *θ*_1_ around the *X*-axis. Therefore, the transformation matrix of coordinate system {1} relative to the base coordinate system {B} is as follows:

(1) $$\begin{array}{c} {\text{⁣}}_{1}^{B}T=\text{Trans}\left(x,l_{\text{unit}}\right)\text{Rot}\left(x,\theta_{1}\right). \end{array}$$

The coordinate system {2*K*} relative to the coordinate system {2*K−*1} can be obtained through the following transformations: first, the coordinate system {2*K*} is initially aligned with the coordinate system {2*K−*1}, then it is rotated *θ*_2*k*_ around the *Z*-axis of {2*K*}. The coordinate system {2*K*+1} relative to the coordinate system {2*K*} can be obtained through the following transformations: first, the coordinate system {2*K*+1} is initially aligned with the coordinate system {2*K*}, then it is translated *l*_unit_ along the *X*-axis of {2*K*+1}, and finally it is rotated *θ*_2*k*+1_ around the *X*-axis of {2*K* + 1}.

Therefore, the transformation matrix between each joint coordinate system is as follows:

(2) $$\begin{array}{c} {\text{⁣}}_{n}^{n-1}T=\left\{\begin{array}{c} \text{Rot}\left(z,\theta \right),n=2,4,6,\ldots \\ \text{Trans}\left(x,l_{\text{unit}}\right)\text{Rot}\left(x,\theta_{n}\right),n=1,3,5,\ldots \end{array}\right.. \end{array}$$

Expanding Equation (2), we can obtain the coordinate system transformation for the first joint module of the snake-like robot:

(3) $$\begin{array}{c} {\text{⁣}}_{B}^{0}T=\left[\begin{array}{cccc} \cos\theta_{1} & -\sin\theta_{1} & 0 & 0\\ \sin\theta_{1} & \cos\theta_{1} & 0 & 0\\ 0 & 0 & 1 & 0\\ 0 & 0 & 0 & 1 \end{array}\right], \end{array}$$

(4) $$\begin{array}{c} {\text{⁣}}_{1}^{B}T=\left[\begin{array}{cccc} 1 & 0 & 0 & l_{\text{unit}}\\ 0 & 1 & 0 & 0\\ 0 & 0 & 1 & 0\\ 0 & 0 & 0 & 1 \end{array}\right]\left[\begin{array}{cccc} 1 & 0 & 0 & 0\\ 0 & \cos\theta_{2} & -\sin\theta_{2} & 0\\ 0 & \sin\theta_{2} & \cos\theta_{2} & 0\\ 0 & 0 & 0 & 1 \end{array}\right]=\left[\begin{array}{cccc} 1 & 0 & 0 & d_{2}\\ 0 & \cos\theta_{2} & -\sin\theta_{2} & 0\\ 0 & \sin\theta_{2} & \cos\theta_{2} & 0\\ 0 & 0 & 0 & 1 \end{array}\right]. \end{array}$$

The coordinate system transformation for the second joint module of the snake-like robot is as follows:

(5) $$\begin{array}{c} {\text{⁣}}_{2}^{1}T=\left[\begin{array}{cccc} \cos\theta_{3} & -\sin\theta_{3} & 0 & 0\\ \sin\theta_{3} & \cos\theta_{3} & 0 & 0\\ 0 & 0 & 1 & 0\\ 0 & 0 & 0 & 1 \end{array}\right], \end{array}$$

(6) $$\begin{array}{c} {\text{⁣}}_{3}^{2}T=\left[\begin{array}{cccc} 1 & 0 & 0 & d_{4}\\ 0 & 1 & 0 & 0\\ 0 & 0 & 1 & 0\\ 0 & 0 & 0 & 1 \end{array}\right]\left[\begin{array}{cccc} 1 & 0 & 0 & 0\\ 0 & \cos\theta_{4} & -\sin\theta_{4} & 0\\ 0 & \sin\theta_{4} & \cos\theta_{4} & 0\\ 0 & 0 & 0 & 1 \end{array}\right]=\left[\begin{array}{cccc} 1 & 0 & 0 & d_{4}\\ 0 & \cos\theta_{4} & -\sin\theta_{4} & 0\\ 0 & \sin\theta_{4} & \cos\theta_{4} & 0\\ 0 & 0 & 0 & 1 \end{array}\right]. \end{array}$$

Since the snake-like robot studied in this paper consists of a total of 11 unit modules, the coordinate system transformation matrix for the *k*-th unit module is as follows:

(7) $$\begin{array}{c} {\text{⁣}}_{2k-2}^{2k-3}T=\left[\begin{array}{cccc} \cos\theta_{2k-1} & -\sin\theta_{2k-1} & 0 & 0\\ \sin\theta_{2k-1} & \cos\theta_{2k-1} & 0 & 0\\ 0 & 0 & 1 & 0\\ 0 & 0 & 0 & 1 \end{array}\right], \end{array}$$

(8) $$\begin{array}{c} {\text{⁣}}_{2k-1}^{2k-2}T=\left[\begin{array}{cccc} 1 & 0 & 0 & d_{2k}\\ 0 & 1 & 0 & 0\\ 0 & 0 & 1 & 0\\ 0 & 0 & 0 & 1 \end{array}\right]\left[\begin{array}{cccc} 1 & 0 & 0 & 0\\ 0 & \cos\theta_{2k} & -\sin\theta_{2k} & 0\\ 0 & \sin\theta_{2k} & \;\cos\theta_{2k} & 0\\ 0 & 0 & 0 & 1 \end{array}\right]=\left[\begin{array}{cccc} 1 & 0 & 0 & d_{2k}\\ 0 & \cos\theta_{2k} & -\sin\theta_{2k} & 0\\ 0 & \sin\theta_{2k} & \;\cos\theta_{2k} & 0\\ 0 & 0 & 0 & 1 \end{array}\right]. \end{array}$$

By multiplying the transformation matrices between the 11 unit modules, we can obtain the transformation matrix from the robot's end effector coordinate system to the global coordinate system {*O*} as follows:

(9) $$\begin{array}{c} {\text{⁣}}_{20}^{0}T={\text{⁣}}_{B}^{0}T{\text{⁣}}_{1}^{B}T{\text{⁣}}_{2}^{1}T\cdot \cdot \cdot {\text{⁣}}_{20}^{19}T=\left[\begin{array}{cccc} n_{x} & o_{x} & a_{x} & p_{x}\\ n_{y} & o_{y} & a_{y} & p_{y}\\ n_{z} & o_{z} & a_{z} & p_{z}\\ 0 & 0 & 0 & 1 \end{array}\right]. \end{array}$$

In Equation (9), *n*_*x*_, *n*_*y*_, *n*_*z*_, *o*_*x*_, *o*_*y*_, *o*_*z*_, *a*_*x*_, *a*_*y*_, *a*_*z*_ represent the orientation of the end effector of the snake-like robot' *p*_*x*_, *p*_*y*_, *p*_*z*_ represent the position coordinates of the end effector of the snake-like robot.

## 3. Snake-Like Robot Workspace Analysis

### 3.1. Traditional Monte Carlo Algorithm for Solving Snake-Like Robot Workspace

The traditional Monte Carlo method is a numerical computation technique based on probability and statistics. Its core idea is to approximate the solution to a problem by generating a large number of random samples and performing statistical analysis. The simple random sampling method is used to randomly select joint angles within the limits of each joint, and then the position of the end effector is solved using the robot's kinematic equations. The more samples are taken, the closer the set of solutions formed by the positions of the end effector will be to the workspace range determined by the robot's structural characteristics. The main steps for solving the snake-like robot workspace using the conventional Monte Carlo method are as follows:

Step 1: Produce randomly sampled values of the snake-like robot joint variables using the random function rand():

(10) $$\begin{array}{c} q_{n}=q_{n}^{\min}+\left(q_{n}^{\max}-q_{n}^{\min}\right)\cdot \mathrm{rand}\left(1,N\right)/N. \end{array}$$

In the above equation, *q* is the joint random variable; *q*^max^ and *q*^min^ are the upper and lower limits of the joint range, and in this paper, we set the joint limit angle of the snake-like robot as ±60°, *n* is the number of robot joints, and *N* denotes the number of samples.

Step 2: The position coordinates *P*_(*x*, *y*, *z*)_ of the end-effector of the snake-like robot are obtained by substituting random values of the robot joint angles into the robot kinematic equations.

Step 3: Using MATLAB simulation software, each position coordinate of the robot is described within the Cartesian coordinate system, and the snake-like robot's workspace is obtained through *N* iterations.

**Figure 4:**
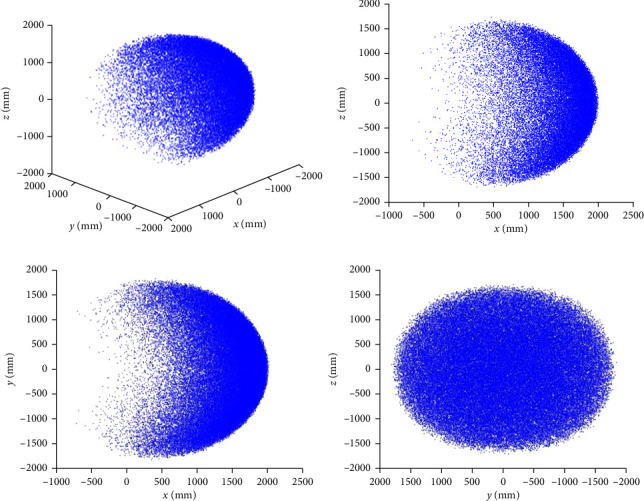
Snake-like robot workspace under randomly distributed sampling: (a) global coordinate system under, (b) *XOZ* plane under, (c) *XOY* plane under, and (d) *YOZ* plane under.

The robot workspace expression obtained in the MATLAB simulation software for *N* = 10^6^ is shown below:

From the results in Figure 4, it can be observed that the robot workspace obtained based on the traditional Monte Carlo method has obvious sparse regions, especially near the boundary of the workspace where the sparsity is large and the description is not accurate enough. This is due to the fact that the principle of the traditional Monte Carlo method is based on the statistical theory of probability, and the simulation for the problem of random features is the basic characteristic of the method. However, the robot workspace is not exactly a random sampling problem in the strict sense by nature. Although increasing the number of sampling points can increase the range of the solution set at the limit position, this will lead to the duplication of points at nonlimit positions, resulting in the waste of points, and also greatly increase the computational cost and fail to achieve the desired effect.

### 3.2. Workspace Solution Based on β Probability Density Distribution Function

Cao et al. [13] used the β probability density distribution function to achieve the purpose of improving the sampling probability of the robot at the limit position. This method is a continuous probability distribution commonly used to describe the distribution of random variables within the interval [0, 1]. Its probability density function can be adjusted by two shape parameters (*α* and *β*) to control the form of the distribution. In spatial sampling, the *β* distribution function is used to increase the sampling probability of the snake-like robot at extreme positions, thereby providing a better description of the workspace boundaries and effectively addressing the issue of sparse point clouds in the robot's workspace boundary.

The expression of the *β* probability density distribution function is shown as follows:

(11) $$\begin{array}{c} f\left(x,\alpha ,\beta \right)=\frac{x^{\alpha -1}{\left(1-x\right)}^{\beta -1}}{\int_{0}^{1}u^{\alpha -1}{\left(1-u\right)}^{\beta -1}du}=\frac{\Gamma \left(\alpha +\beta \right)}{\Gamma \left(\alpha \right)\Gamma \left(\beta \right)}x^{\alpha -1}{\left(1-x\right)}^{\beta -1}=\frac{1}{B\left(\alpha ,\beta \right)}x^{\alpha -1}{\left(1-x\right)}^{\beta -1}, \end{array}$$

**Figure 5:**
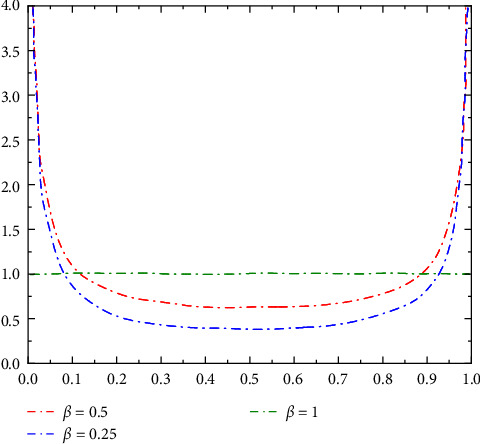
Probability density function curve of *β* distribution.

In the equation shown, Γ(*z*) is the gamma function. The random variable *X* obeys the *B* distribution with parameters *α* and *β* usually written as *X* ~ *Be*(*α*, *β*). The function curves for *β* = 0.25, *β* = 0.5, and *β* = 1 are shown in Figure 5.

**Figure 6:**
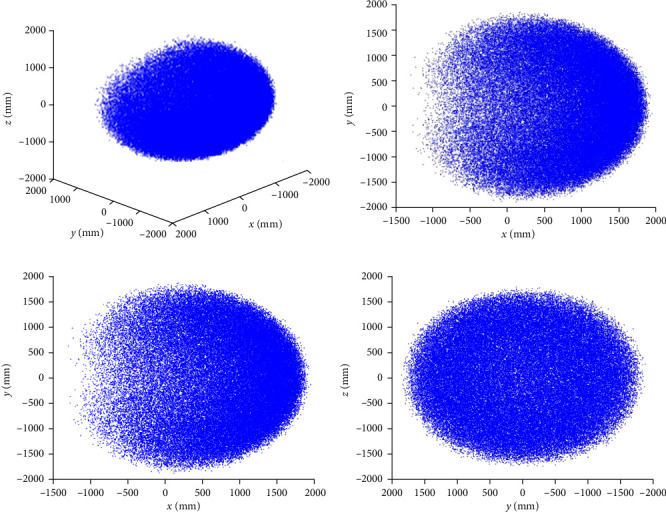
Workspace under the probability density function of the *β* distribution: (a) under the global coordinate system, (b) under the *XOZ* plane, (c) under the *XOY* plane, and (d) under the *YOZ* plane.

In solving the snake-like robot's workspace using the traditional Monte Carlo algorithm, the importance sampling method is employed to improve estimation accuracy by altering the probability distribution *β*. As shown in Figure 5, by selecting *β* = 0.25 and weighting the samples, sampling errors in low-probability regions are reduced; let the number of samples *N* = 10^6^; the workspace of the snake-like robot, obtained through MATLAB simulation software, is shown in Figure 6.

Based on the results in Figure 6, by comparing it with the robot workspace obtained using the traditional Monte Carlo method, the robot workspace obtained by sampling with *β* distribution is better to improve the density distribution of points at the boundary of the workspace and better to express the real range of the limit position of the snake-like robot in the process of motion. However, we can see that the robot workspace still has the problems of rough boundary and “noisy,” so it is necessary to further improve the accuracy.

### 3.3. Workspace Solution Based on Gaussian Probability Density Distribution Function

To address the issues of “high noise” and insufficient accuracy in the boundary of the snake-like robot's workspace, this paper proposes an improved Monte Carlo method for solving the robot's workspace. This method is based on the Gaussian distribution probability density function to solve the snake-like robot's workspace. The Gaussian distribution is a common probability distribution, and its probability density function is determined by the mean and standard deviation. In spatial sampling, the Gaussian distribution achieves more accurate sampling due to its concentration around the mean point. By dynamically adjusting the standard deviation of the Gaussian distribution, the density of the sampled points can be controlled, thereby improving the accuracy of the workspace boundary description. The main steps are as follows:

Step 1: Generate a seed workspace.

Using the traditional Monte Carlo method, sample the robot *N*_1_ times to obtain the end effector coordinate value *P*_(*x*, *y*, *z*)_. Find the maximum and minimum values of the robot's workspace points on the three coordinate axes, which are *x*_max_, *y*_max_, *z*_max_ and *x*_min_, *y*_min_, *z*_min_. Then follow these steps:

1. Generate a cube that envelops the seed workspace, and add a value *Δ* in each direction of the coordinate axis, and then the vertex coordinates of the cube are $\left(x_{\min}-\frac{\Delta }{2},y_{\min}-\frac{\Delta }{2},z_{\min}-\frac{\Delta }{2}\right)$. Among them,

(12) $$\begin{array}{c} \Delta =\frac{\left(\left|x_{\max}-x_{\min}\right||+|\left|y_{\max}-y_{\min}\right||+|\left|z_{\max}-z_{\min}\right|\right)}{12}, \end{array}$$

and,

(13) $$\begin{array}{c} \left\{\begin{array}{c} l=\left|x_{\max}-x_{\min}\right|+\Delta \\ w=\left|y_{\max}-y_{\min}\right|+\Delta \\ h=\left|z_{\max}-z_{\min}\right|+\Delta \end{array}\right.. \end{array}$$

In the formula, *l*, *w*, and *h* are the length, width, and height of the cube.

2. Divide a cube into *k* = *n*_*x*_ × *n*_*y*_ × *n*_*z*_ smaller cubes of equal size along the *x*, *y*, and *z* directions. Then traverse the number of points contained in each small cube. In order to ensure that each location can be accurately described, a precision threshold *N*_p_ is given.
3. Find a cube with a point count less than *N*_p_, establish a database Database_1, and store the cube in the database. The number of points contained in each small cube is recorded as *N*_c_ (*c* = *m*,…,*n*), among them (1 ≤ *m*, *n* ≤ *k*).
4. Find each point in database Database_1 and create a database Database_2 to store the values of these points.

**Figure 7:**
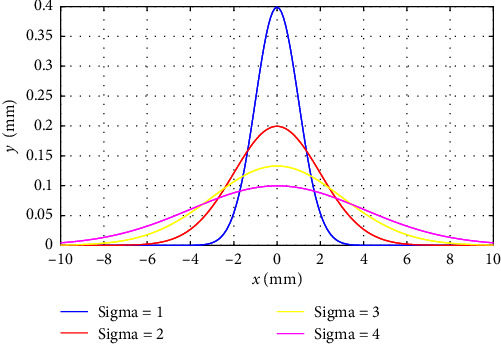
Gaussian distribution probability density function curve.

Step 2: The Gaussian distribution probability density function is used to expand the sampling of the robot's seed workspace. In this process, based on the sampling characteristics of the Gaussian distribution, a precision control threshold is set, and the standard deviation of the Gaussian distribution is dynamically adjustable to ensure that the position of each point in the robot's workspace is accurately described. The Gaussian distribution probability density function is shown in Figure 7.

Step 3: Growing the small cubes in database Database_1 with *n*_*c*_ = |*N*_*p*_ − *N*_*c*_| for each cube, the robot's workspace is obtained through repeated adjustments.

**Figure 8:**
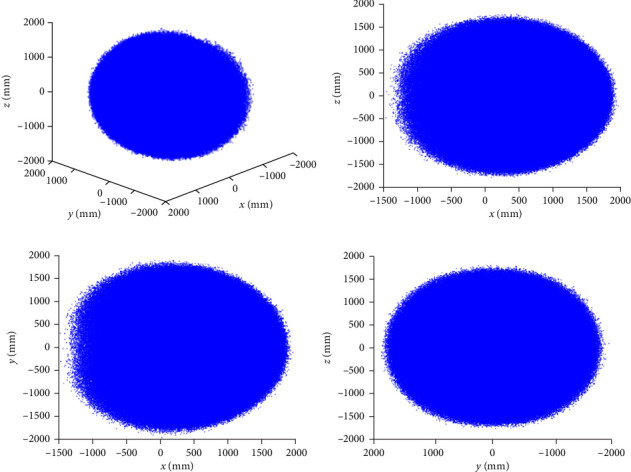
Improvements have been made to the workspace obtained through the Monte Carlo method for the snake-like robot: (a) under the global coordinate system, (b) under the *XOZ* plane, (c) under the *XOY* plane, and (d) under the *YOZ* plane.

Taking *N* = 10^6^, the workspace of the snake-like robot is generated using the method based on the sampling of *β* distribution and the expansion of the seed workspace by Gaussian distribution, respectively, and a comparison of the results is shown in Figure 8.

**Figure 9:**
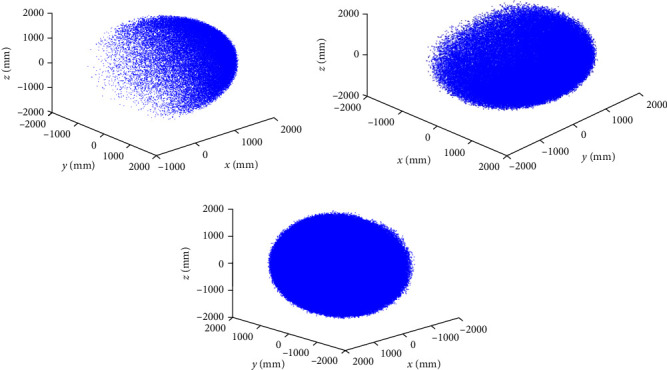
The workspaces obtained through the three methods for solving the snake-like robot are as follows: (a) traditional Monte Carlo method, (b) *β* distribution sampling method, and (c) improved Monte Carlo method.

When using a total of *N* = 10^6^ randomly sampled points, the workspace of the snake-like robot obtained through the traditional Monte Carlo method, the *β* distribution sampling method, and the improved Monte Carlo method proposed in this paper are shown in Figure 9. The time taken for obtaining the workspace using each of these three methods is then statistically analyzed, as presented in Table 2.

As shown in Figure 9, under the same random sampling points, the scatter distribution of the snake-like robot's workspace generated by the improved Monte Carlo method is more uniform, making it more suitable for solving the snake-like robot's workspace. According to Table 2, it is noted that the improved Monte Carlo method takes a longer time to compute the robot's workspace.

To quantitatively describe the more uniform scatter distribution of the snake-like robot's workspace generated by the improved Monte Carlo method, information entropy is used to evaluate the uniformity of the scatter distribution in the point clouds obtained from the traditional Monte Carlo method, *β* distribution, and Gaussian distribution.

Information entropy, as a statistical indicator, quantifies the uncertainty or randomness of the point cloud distribution. The lower the value of information entropy, the more uneven the point cloud distribution is, and the higher the value of information entropy, the more uniform the distribution is. The point cloud data of the snake-like robot's workspace obtained using the traditional Monte Carlo method, *β* distribution, and Gaussian distribution are divided into multiple small grid cells. The number of points in each grid cell represents the point density in that region. The probability for each grid is calculated based on the number of points in it, defined as the ratio of the points in the grid to the total number of points. The information entropy formula is as follows:

(14) $$\begin{array}{c} H\left(X\right)=-\sum_{i=1}^{n}p\left(x_{i}\right)\log\left(p\left(x_{i}\right)\right). \end{array}$$

In the formula, *p*(*x*_*i*_) is the probability of the *i*-th grid cell, and *n* is the total number of grid cells.

**Figure 10:**
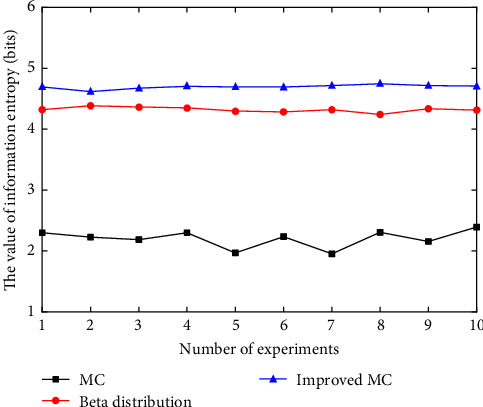
Information entropy of the robot's workspace point cloud results under 10 experimental trials.

Ten samples were taken for each of the three methods to obtain the point cloud maps of the robot's workspace. The information entropy of the point cloud for each experimental result was then calculated, and the statistical results are shown in Figure 10 (MC stands for the traditional Monte Carlo method.).

The average information entropy of the point cloud for the snake-like robot's workspace obtained from 10 experimental trials using the traditional Monte Carlo method, *β* distribution, and Gaussian distribution is shown in Table 3.

As shown in Table 3, the average information entropy of the point cloud generated by the improved Monte Carlo method is the highest. Therefore, the point cloud distribution of the snake-like robot's workspace generated by the improved Monte Carlo method is more uniform, making it more suitable for solving the workspace of the snake-like robot.

## 4. Volume Finding of the Working Space of the Snake-Like Robot

The workspace represents the range of robot motion, and the size of the workspace can be measured in terms of volume. Accurate calculation of the volume of robot workspace is important for robot motion control, trajectory planning, and structural parameter design optimization. In this section, the volume of the robot's workspace is obtained by using the boundary extraction method based on algorithm *α* − shape for the results obtained by expanding the seed workspace based on Gaussian distribution in the previous section.

### 4.1. *α* − *shape* Principle of the Algorithm

**Figure 11:**
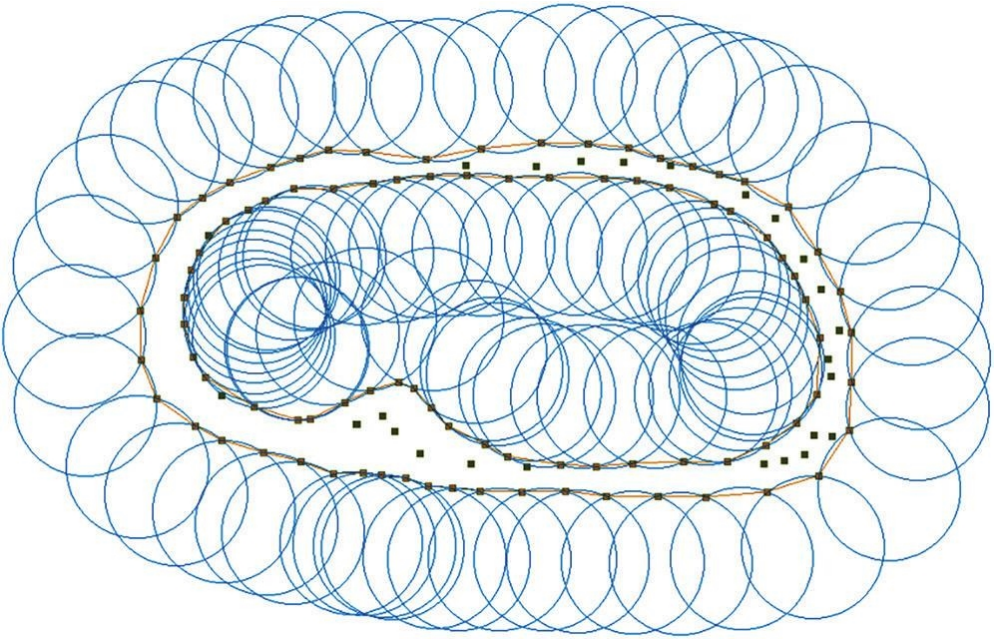
Schematic diagram of the algorithm.

The *α* − shape algorithm can perform edge extraction on a bunch of disordered discrete points while obtaining a reconstructed graph with a polygon boundary for a 2D point cloud and a polyhedron boundary for a 3D point cloud. The principle is that in a finite discrete point set *S*, there are *n* points composed of *n* points, which can form *n*^*∗*^ (*n–*1) line segments, we can determine which line segment is the one on the boundary line by the following method: within the plane point set *S*, draw a circle of radius over any two points *P*_1_ and *P*_2_ (obviously, there should be two circles over the determined two points at a given radius); if there is no other point in this circle, then consider points *P*_1_ and *P*_2_ which are boundary points and their connecting line *P*_1_*P*_2_ is the boundary line segment. Therefore, when the value of *α* is very small, every point is on the boundary; if *α* tends to positive infinity, the boundary line formed is a convex package of the point set *S*. The result of its implementation is shown in Figure 11.

Similarly, in 3D space, the *α* − shape algorithm discriminates the boundary by making a ball of radius *α* at three points in the point set *S*. From this, a triangular region is created at the boundary point, and the surface of the discrete points is reconstructed in the form of a triangular region. The result changes with the value of *α*. A large value of *α* will retain more triangular regions and show a convex packet of the point set, and a small value of *α* will reconstruct the surface is too small, the reconstructed surface becomes vacant. At *α* = 0, the surface degenerates to the original point set.

### 4.2. Volume Calculation of the Snake-Like Robot Workspace

**Figure 12:**
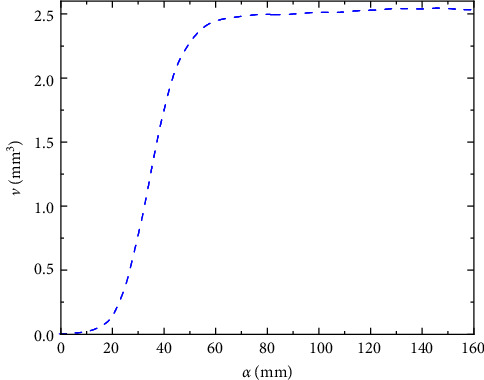
Volume versus parameter relationship.

The workspace of the robot in this paper is similar to a sphere, and the reconstructed result of its 3D point cloud should also be represented as a closed space of certain shape. Therefore, its volume can be calculated using the volume() function in MATLAB. The *α* − shape algorithm can express the point cloud shape visually, and its accuracy is determined by the *α* value. If the *α* is too small, the reconstructed surface of the robot workspace will have more vacancies and behave as some discrete triangular regions, and the volume cannot be found at this time. When *α* exceeds a certain value, the reconstructed surface of the robot workspace is saturated, and the volume will not change significantly with the value of *α*, and the reconstruction effect reaches the best at this time. In order to determine the appropriate value of *α*, the data within the interval (0.160) are selected to calculate the volume of the robot workspace in this paper, and the results are shown in Figure 12.

**Figure 13:**
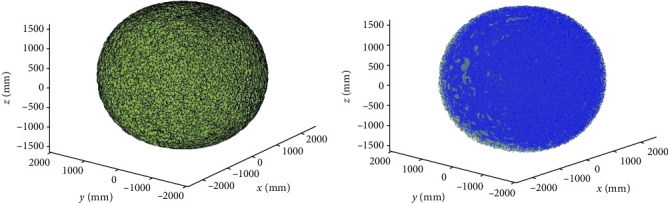
Workspace reconstruction results: (a) *α* = 80 reconstruction results and (b) comparison of reconstructed surface and point cloud.

It can be seen from the figure that after *α* = 80, the volume no longer changes significantly, so the optimal parameter is *α* = 80, when the volume is *V* = 2.581 × 10^9^mm^3^, and the reconstructed surface of the robot workspace is shown in Figure 13a. In order to verify the reconstruction accuracy of the *α* − shape algorithm, the reconstructed surface is compared with the snake-like robot workspace point cloud, and the results are shown in Figure 13b. From the graphical results, we can see that the blue point clouds are evenly distributed on the surface, indicating that the algorithm has high accuracy.

## 5. Conclusion

1. This paper proposes a Gaussian-enhanced Monte Carlo method for solving the workspace of a snake-like robot designed for operations along high-voltage transmission cables. The mechanical structure of the snake-like robot is introduced, and a kinematic model is established based on the structural characteristics of the robot. The pose coordinate transformation relationships between various joint modules of the robot are described.
2. An improved Monte Carlo method is introduced for obtaining the workspace of the snake-like robot. The obtained workspace using this method is compared and analyzed against workspaces obtained through traditional Monte Carlo method and a method based on *β* probability density function distribution sampling. The results indicate that, with the same number of randomly sampled points, the improved Monte Carlo method produces a smoother and more accurate boundary for the snake-like robot's workspace.
3. The *α* − shape algorithm is employed to extract the point cloud boundary of the snake-like robot's workspace and calculate its volume. The volume calculation value is used as a reference to measure the reconstruction effectiveness. A method for parameter *α* determination based on volume values is provided, yielding satisfactory reconstruction results.

## Figures and Tables

**Table 1:** Kinematic parameters for snake-like robot.

Serial number	*θ*/rad	*d*/mm	*a*/mm	*α*/rad	Value range of *θ*/rad
1	*θ*_1_	128	0	*π*/2	[−*π*/3, *π*/3]
2	*θ*_2_	0	0	−*π*/2	[−*π*/3, *π*/3]
3	*θ*_3_	128	0	*π*/2	[−*π*/3, *π*/3]
4	*θ*_4_	0	0	−*π*/2	[−*π*/3, *π*/3]
5	*θ*_5_	128	0	*π*/2	[−*π*/3, *π*/3]
6	*θ*_6_	0	0	−*π*/2	[−*π*/3, *π*/3]
7	*θ*_7_	128	0	*π*/2	[−*π*/3, *π*/3]
8	*θ*_8_	0	0	−*π*/2	[−*π*/3, *π*/3]
9	*θ*_9_	128	0	*π*/2	[−*π*/3, *π*/3]
10	*θ*_10_	0	0	−*π*/2	[−*π*/3, *π*/3]
11	*θ*_11_	128	0	*π*/2	[−*π*/3, *π*/3]
12	*θ*_12_	0	0	−*π*/2	[−*π*/3, *π*/3]
13	*θ*_13_	128	0	*π*/2	[−*π*/3, *π*/3]
14	*θ*_14_	0	0	−*π*/2	[−*π*/3, *π*/3]
15	*θ*_15_	128	0	*π*/2	[−*π*/3, *π*/3]
16	*θ*_16_	0	0	−*π*/2	[−*π*/3, *π*/3]
17	*θ*_17_	128	0	*π*/2	[−*π*/3, *π*/3]
18	*θ*_18_	0	0	−*π*/2	[−*π*/3, *π*/3]
19	*θ*_19_	128	0	*π*/2	[−*π*/3, *π*/3]
20	*θ*_20_	0	0	−*π*/2	[−*π*/3, *π*/3]

**Table 2:** Comparison of the time taken for obtaining the workspace of the snake-like robot using the three different methods.

Method	Traditional Monte Carlo method	*β* distribution sampling method	Improved Monte Carlo method
Time/s	25.91	78.90	297.51

**Table 3:** Average information entropy of the robot's workspace point cloud results under 10 experimental trials.

Method	Traditional Monte Carlo method	*β* distribution sampling method	Improved monte carlo method
The value of information entropy/bits	2.3	4.2	4.6

## Data Availability

The data used to support the findings of this study are available from the corresponding author upon request.
